# Antibiotics Use in the Treatment of Patients With Appendicitis in Three Hospitals in Taif City, Kingdom of Saudi Arabia: A Retrospective Study

**DOI:** 10.7759/cureus.83139

**Published:** 2025-04-28

**Authors:** Hatim A Elsheikh, Amal I Elsiddig, Nouf A Alqarni, Amnah Dobel, Batool Y Asiri, Shaden O Alqurashi, Mohamed Alsaeed

**Affiliations:** 1 Department of Pharmacology, College of Medicine, Taif University, Taif, SAU; 2 Department of Surgery, College of Medicine, Taif University, Taif, SAU; 3 College of Medicine, Taif University, Taif, SAU

**Keywords:** abdominal, acute appendicitis, antibiotics, hospital stays, nonoperative, surgery

## Abstract

Introduction: Acute appendicitis is a common surgical emergency caused by inflammation and infection of the appendix, yet there is limited consensus on its management despite its widespread prevalence. While surgery remains the standard, the role of antibiotics, particularly in nonoperative management, is increasingly recognized. This study aims to identify the antibiotic regimens for appendicitis in three governmental and military hospitals in Taif City, assess their consistency with international guidelines, review effectiveness, and determine the length of hospital stay for patients following different treatment methods.

Method: A retrospective chart review design was used to analyze the antibiotics regimen used for managing appendicitis and perforated appendicitis among patients of various age groups in Taif City. Data was collected from the medical records of patients admitted to pediatric and adult surgery units from January 2020 to December 2022. SPSS software, version 26.0, was used for descriptive analysis, while inferential statistical tests were used to compare hospital stays.

Results: The study analyzed 646 hospital records for patients with appendicitis. Surgical interventions were common, with open appendectomy being the most common procedure, 522 (80.8%). Only 25 (3.9%) patients received preoperative antibiotics, while 481 (74.5%) patients received postoperative antibiotics, mostly Augmentin, with oral administration being preferred in 450 (69.7%) cases. Postoperative outcomes showed low complication rates, with only 54 (8.4%) experiencing surgical-site infections, hernia, and minimal abdominal pain. The mean hospital stay was 2.2 days.

Conclusion: The study suggests that compliance with preoperative antibiotic guidelines should be improved, and nonoperative management options should be discussed in certain patient populations. The focus should be on rational antibiotic usage and patient-oriented treatment methods to maximize appendicitis treatment compared to existing literature.

## Introduction

Appendicitis is a common emergency surgical condition, with an estimated risk of 8.6% in men and 6.7% in women [1]. The incidence of appendicitis, for instance, in 2023, for Western countries was observed as 100 per 100,000 persons, and the increasing trend in developing countries makes appendectomy the most frequent surgical procedure [1,2]. Acute appendicitis can be uncomplicated or complicated, with uncomplicated appendicitis treated mainly by appendectomy [3,4]. Nonoperative intervention, such as intravenous antibiotics, is preferred for complicated cases. Antibiotic therapy has also been successfully used as an alternative for uncomplicated appendicitis [5].

Surgical intervention was deemed imperative for early disease clearance. Additionally, appendectomy offers the benefits of a single hospital admission and the prevention of the recurrence of the pathological issue [6]. However, the procedure has several complications such as surgical-site infections, bowel obstructions, adhesions, colonic fistulas, and hematomas [7,8]. Nonoperative treatment is preferred in dealing with complicated acute appendicitis because it eliminates irreversible loss of organ functionality, in addition to lower costs and the advantages of eliminating the risk factors associated with invasive surgical techniques [9,10]. 

The need for surgery has been challenged in adults and children, and the COVID-19 pandemic emphasized the role of antibiotics in treating appendicitis [11]. Nonetheless, broad-spectrum antibiotic therapy should be administered within 1 hour before an appendectomy to minimize the risk of infectious complications [12]. The World Society of Emergency Surgery recommends a single dose of antibiotics before skin incision to reduce the risk of wound infection and intra-abdominal abscess, aligning with the American Pediatric Surgical Association's recommendation regarding the use of preoperative prophylactic antibiotics in pediatric patients undergoing appendectomy [13]. The England National Health Service (NHS) recommends administering gentamycin and metronidazole preoperatively in all lower gastrointestinal surgeries [14,15]. Moreover, previous trials recommend starting antibiotics for acute appendicitis with an intravenous antibiotic for 1-3 days, followed by oral antibiotics for 7-10 days [16]. The combination of long-acting antibiotics, such as ceftriaxone, with metronidazole has been demonstrated to facilitate early recovery and expedite hospital discharge [17,18]. In addition, administration of broad-spectrum antibiotics preoperatively in patients with appendicitis has decreased the incidence of complications, such as wound infections and abscess formation, and is sufficient for nonperforated appendicitis, while postoperative broad-spectrum antibiotics are necessary in cases of perforated appendicitis [19,20]. Furthermore, clinical studies confirmed that antibiotics have helped over 70% of appendicitis patients avoid surgery, saving time and reducing complications [21,22]. 

Studies on the use and effectiveness of antibiotic regimens in the management of appendicitis patients in Taif hospitals and the comparison of their compliance with international standards are scarce. The purpose of this study is to identify the antibiotic regimens used for appendicitis in three hospitals in Taif City, to determine whether these regimens are consistent with international guidelines, and to assess the effectiveness, response, and length of hospital stay associated with the regimens employed.

## Materials and methods

Study design

This study employed a retrospective chart review research design to analyze the antibiotic regimen used for managing appendicitis among patients of different age groups in Taif. The data were collected from patients’ medical records, including those who underwent surgery and received antibiotics for appendicitis in the pediatric and adult surgery units from January 2020 to December 2022, while ensuring the confidentiality of these data. Using the case notes available in the clinical setting, the study aimed to assess the compliance of antibiotics administered in accordance with international guidelines, their effectiveness, and their impact on the length of hospital stay.

Inclusion criteria

All medical records of patients from all age groups who had been admitted to the hospital with appendicitis during the period from January 2020 to December 2022. 

Exclusion criteria

Medical records of appendicitis patients with missing or incomplete data. 

Sample size

The study included 646 records of all patients with appendicitis admitted to the three selected (governmental and military) hospitals in Taif City, Saudi Arabia. Of them, 397 were male patients, and 249 were female patients.

Data collection

The study is a retrospective review of medical records from patients with appendicitis from three selected hospitals in Taif City. A structured data collection sheet was developed to capture sociodemographic information and variables related to the surgical condition, including clinical diagnosis, types and regimens of antibiotics used, administration routes, treatment durations, and lengths of hospitalization. The surgical procedure and complications were also included. Trained research personnel systematically extracted relevant data from patient charts, ensuring accuracy and consistency. The data collection process adhered to ethical guidelines and maintained patient confidentiality.

Data analysis

Data analysis was performed using Statistical Package for the Social Sciences (SPSS) software, version 26.0 (IBM Corp, Armonk, NY). All analyses presented in this paper were conducted at the 0.05 level of significance, utilizing both descriptive and inferential statistics. Quantitative data provided descriptive analysis of demographic information, the surgical interventions performed on the participants, and the details on the outcomes of the surgeries. Descriptive analysis involved the use of inferential statistical tests, such as one-way analysis of variance (ANOVA), to compare hospital stays among the different study groups according to the type of antibiotic used: none, a single antibiotic, or multiple antibiotics. Additionally, to compare measures between groups, the post-hoc test was used, specifically the Dunnett T3 test. The independent-sample t-test and chi-square test were applied to determine the significance and relationships between variables. The significance level of the test was set at p <0.05, which was used to determine the level of statistical significance for testing the hypothesis.

Ethical consideration

Ethical approval for this study was obtained from the Institutional Review Board (IRB) of the Research and Studies Department, Directorate of Health Affairs - Taif, Ministry of Health (Reference number: HAP-02-T-067-795) and the Research Ethics Committee of Armed Forces Hospitals, Western Region, Taif, Saudi Arabia (Reference number: H-02-T-078-2024-937). The study was initiated after obtaining permission from the administrations of the two relevant government hospitals (King Faisal Medical Complex and King Abdulaziz Specialist Hospital) and Alhada Armed Forces Hospital in Taif to conduct the retrospective patient chart review using their computerized hospital administrative registration systems. The study adhered to ethical principles, ensuring the confidentiality of patient data. Patient identities were anonymized in the data analysis process to protect privacy and confidentiality. Ethical considerations also included obtaining permissions for data access and ensuring compliance with institutional policies and regulations regarding research involving human subjects.

## Results

This study included 646 participants. Table 1 presents the various demographic parameters of the participants. The mean age is 23.9 years, with a standard deviation of 12.9 years, indicating a relatively diverse age distribution. Most of the participants, 454 (70.3%), fall within the 12-39-year age range, while 20.3% are less than 12 years old, and a smaller proportion (8.0% and 1.4%) are in the 40-59-year and 60 years or more age groups, respectively. This age distribution suggests that the study population is predominantly composed of younger individuals, which may reflect the specific medical conditions or healthcare-seeking patterns observed in this study. Regarding sex distribution, most participants were males, 397 (61.5%), while females comprised only 249 (38.5%), suggesting a possible imbalance in the sex distribution of the patients. This finding may be useful in the epidemiological or clinical approach to the medical conditions in question, as documented differences in disease rates or symptom manifestation exist between male and female populations. Regarding the diagnostic categories, 608 (94.1%) participants were diagnosed with acute appendicitis, while one (0.15%), 36 (5.6%), and one (0.15%) presented with appendicular mass, complicated acute appendicitis, and undifferentiated abdominal pain, respectively. Altogether, the sociodemographic and diagnostic data presented in this work are sufficient to provide an understanding of the sample, which can be used to frame the context for interpreting the study's outcomes and for further research in the field.

**Table 1:** Sociodemographic Characteristics of Participants (n=646)

Parameter	Description	Number	Percent
Age	Less than 12 years	131	20.3
	12 to 39 years	454	70.3
	40 to 59 years	52	8
	60 years or more	9	1.4
Sex	Female	249	38.5
	Male	397	61.5
Diagnosis	Acute appendicitis	608	94.1
	Appendicular mass	1	0.15
	Complicated acute appendicitis	36	5.6
	Undifferentiated abdominal pain	1	0.15

The overall number of patients who underwent surgery was 646; the most common type of surgical intervention was open appendectomy, 522 (80.8%). Despite the high number of surgical patients, only 25 (3.9%) of them received preoperative antibiotics, while cefuroxime was the most frequently administered antibiotic, at 10 (1.5%). The route of preoperative antibiotics was mainly IV, though a small proportion (0.6%) received oral antibiotics. The other parameter that revealed variability was the duration of the preoperative antibiotics; the majority of patients, 621 (96.1%), didn’t receive antibiotics preoperatively, while the rest received them for as few as one day or as long as seven days (Table 2). 

**Table 2:** Parameters Related to Surgery and Preoperative Antibiotics (n=646)

Parameter	Description	Number	Percent
Surgery was performed	Yes	646	100
Type of surgery	Laparoscopic appendectomy	124	19.2
	Open appendectomy	522	80.8
Preoperative antibiotics	No	621	96.1
	Yes	25	3.9
Preoperative antibiotics name	Augmentin	3	0.5
	Cefazolin	6	0.9
	Ceftriaxone	6	0.9
	Cefuroxime	10	1.5
	Not given preoperative antibiotic	621	96.1
Route of preoperative antibiotics	IV	20	3.1
	IV and IM	1	0.2
	Oral	4	0.6
	Not given preoperative antibiotic	621	96.1
Duration of preoperative antibiotics (days)	1	8	1.2
	2	-	-
	3	10	1.5
	4	2	0.3
	5	3	0.5
	7	2	0.3
	Not given preoperative antibiotic	621	96.1

The types and duration of postoperative antibiotic regimens in a total of 646 patients are displayed in Table 3. The majority of patients, 481 (74.5%), in the study were administered postoperative antibiotics with a single-type regimen, 461 (71.4%). Of the various antibiotics used specifically, Augmentin was the most frequently administered, either alone, 259 (40.0%), or in combination with other antibiotics, and cefuroxime was the second most common, 162 (25.1%). Among the administered postoperative antibiotics, the majority of patients received the doses orally, 450 (69.7%), which demonstrates that oral administration is the preferred route over IV or IM administration. The periods of antibiotic administration also differed, with the highest rate of 258 (39.9%) completing their courses within five days. 

**Table 3:** Participants' Postoperative Antibiotics (n=646)

Parameter	Description	Number	Percent
Postoperative antibiotics	No	165	25.5
	Yes	481	74.5
Regimen of antibiotics	Single	461	71.4
	Multiple	20	3.1
	Not given postoperative antibiotic	165	25.5
Name of postoperative single antibiotics	Amoxicillin	2	0.3
	Augmentin	259	40.0
	Cefalexin	1	0.2
	Cefdinir	3	0.5
	Ceftriaxone	1	0.2
	Cefuroxime	162	25.1
	Ciprofloxacin	24	3.7
	Gentamicin	1	0.2
	Levofloxacin	1	0.2
	Metronidazole	6	0.9
	Tazobactam	1	0.2
	Total number of patients given a single antibiotic	461	71.4
Name of postoperative multiple antibiotics	Augmentin + ciprofloxacin	1	0.2
	Augmentin + metronidazole	7	1.1
	Cefuroxime + metronidazole	12	1.9
	Total number of patients given multiple antibiotics	20	3.1
Route of postoperative antibiotics	IV	29	4.5
	IV and IM	1	0.2
	Oral	450	69.7
	Oral and IV	1	0.2
	Not given postoperative antibiotic	165	25.5
Duration of postoperative antibiotics	1 to 4 days	39	6.2
	5 days	258	39.9
	6 to 7 days	171	26.5
	More than 7 days	13	2.1

Based on the data provided in Table 4 regarding the postoperative status of the patients, the majority did not develop complications, with 52 (8.0%) developing a surgical-site infection and two (0.4%) patients reporting abdominal pain and hernia. Similarly, Table 4 shows that 582 (90.1%) patients are discharged within the first three days, with a mean hospitalization period of 2.2 days.

**Table 4:** Participants' Postoperative Complications (n=646) STD, standard deviation of the mean.

Parameter	Description	Number	Percent
Complications	Abdominal pain	1	0.2
	Hernia	1	0.2
	Surgical-site infection	52	8
	No complications	592	91.6
Length of hospital stay (days) (mean = 2.2, STD=1.6)	1 day or less	239	37
	2 or 3 days	343	53.1
	More than 3 days	64	9.9
Vomiting before surgery	No	356	55.1
	Yes	290	44.9
Vomiting after surgery	No	643	99.5
	Yes	3	0.5
Fever before surgery	No	532	82.4
	Yes	114	17.6
Fever after surgery	No	637	98.6
	Yes	9	1.4
Abdominal pain after surgery	No	622	96.3
	Yes	24	3.7

The effect of antibiotics regimen on length of hospital stay has a statistically significant relationship with a p-value of less than 0.001; the mean length of stay of patients who did not receive antibiotics was 1.62 days compared to those who received a single antibiotic, which was 2.42 days, while multiple antibiotics average hospital stay was 2.0 days (Table 5).

**Table 5:** Effect of Antibiotic Regimens on Patients' Length of Hospital Stay *One-way ANOVA. ANOVA, analysis of variance; STD, standard deviation of the mean.

Parameter	None	Single	Multiple	p-Value*
Length of stay (mean ± STD)	1.62±1.66	2.42±1.57	2.0±0.21	<0.001

Table 6 presents the post-hoc analysis (Dunnett's T3) of the effect of antibiotic regimen on patients’ length of hospital stay, which was significant when comparing a single antibiotic regimen to no antibiotic treatment (p-value=0.001). It also shows no relationship between multiple antibiotic regimens and not taking any antibiotics.

**Table 6:** Post-hoc (Dunnett's T3) Test of the Effect of Antibiotic Regimens on Patients' Length of Hospital Stay *Post-hoc (Dunnett's T3) test.

Parameter	p-Value*
No antibiotic vs single antibiotic	<0.001
No antibiotic vs multiple antibiotics	0.604

Antibiotic use has a statistically significant relationship with the type of surgery (p-value=0.029), length of hospital stays (p-value=0.0001), and vomiting after management (p-value=0.015). It also shows no relationship to vomiting before management, fever before and after management, and abdominal pain after management (Table 7).

**Table 7:** Relation Between Antibiotic Regimen and Management Outcome *p-Value was considered significant if ≤0.05. Chi-square test was used. N/A, not applicable.

Parameter	Description	Antibiotic regimen			Total (N=646)	p-Value*
		None	Single	Multiple		
Surgery performed	Yes	165	461	20	646	N/A
	%	100	100	100	100	
Diagnosis	Acute appendicitis	151	438	19	608	N/A
	%	91.5	95.0	95.0	94.1	
	Appendicular mass	0	1	0	1	
	%	0.0	0.2	0.0	0.2	
	Complicated appendicitis	13	22	1	36	
	%	7.9	4.8	5.0	5.6	
	Undifferentiated abdominal pain	1	0	0	1	
	%	0.6	0.0	0.0	0.2	
Type of surgery	Open appendectomy	139	364	19	522	0.029
	%	84.2	78.9	95.0	80.8	
	Laparoscopic appendectomy	26	97	1	124	
	%	15.8	21.0	5.0	19.2	
Antibiotic before surgery	No	621	0	0	621	N/A
	%	96.1	0.0	0.0	96.1	
	Yes	0	25	0	25	
	%	0.0	3.9	0.0	3.9	
Duration of postoperative antibiotics	1-4 days	0	38	1	39	N/A
	%	0.0	8.2	5.0	6.0	
	5 days	0	247	11	258	
	%	0.0	53.6	55.0	39.9	
	6 to 7 days	0	165	6	171	
	%	0.0	35.8	30.0	26.5	
	More than 7 days	0	11	2	13	
	%	0.0	2.4	10.0	2.0	
Postoperative complications	Abdominal pain	0	1	0	1	N/A
	%	0.0	0.2	0.0	0.2	
	Hernia	0	1	0	1	
	%	0.0	0.2	0.0	0.2	
	Surgical-site infection (SSI)	11	41	0	52	
	%	6.7	8.9	0.0	8.0	
	None	154	418	20	592	
	%	93.3	90.8	100.0	91.6	
Length of hospital stay	1 day	109	120	6	235	0.0001
	%	66.1	26.0	30.0	36.4	
	2 or 3 days	41	293	12	346	
	%	24.8	63.6	60.0	53.4	
	More than 3 days	15	48	2	65	
	%	9.1	10.4	10.0	10.1	
Vomiting before	No	85	261	10	356	0.525
	%	51.5	56.6	50.0	55.6	
	Yes	80	200	10	290	
	%	48.5	43.4	50.0	44.4	
Vomiting after	No	162	461	20	643	0.015
	%	98.3	100.0	100.0	99.5	
	Yes	3	0	0	3	
	%	1.8	0.0	0.0	0.5	
Fever before	No	127	386	19	532	0.223
	%	77.0	83.7	95.0	82.4	
	Yes	38	75	1	114	
	%	23.0	16.3	5.0	17.6	
Fever after	No	165	452	20	637	0.155
	%	100.0	98.1	100.0	98.6	
	Yes	0	9	0	9	
	%	0.0	1.9	0.0	1.4	
Abdominal pain after	No	159	443	20	622	0.688
	%	96.3	96.1	100.0	96.3	
	Yes	6	18	0	24	
	%	3.7	3.9	0.0	3.7	

Table 8 shows a statistically significant relationship between performing surgery and antibiotic regimen (p-value=0.027), diagnosis (p-value=0.0001), taking preoperative antibiotics (p-value=0.0001), and the duration of postoperative antibiotics (p-value=0.011). On the other hand, it shows no relationship with the dosage of postoperative antibiotics, length of stay in the hospital, and vomiting and fever before management.

**Table 8:** Relationship Between Performing Surgery and Management Outcomes *p-Value was considered significant if ≤0.05. Chi-square test was performed. N/A, not applicable.

Parameter	Description	Surgery		p-Value*
		Number	%	
Antibiotic regimen	None	165	25.5	0.027l
	Single	461	71.4	
	Multiple	20	3.1	
Diagnosis	Acute appendicitis	608	94.1	0.0001
	Appendicular mass	1	0.2	
	Complicated acute appendicitis	36	5.6	
	Undifferentiated abdominal pain	1	0.2	
Type of surgery	Open appendectomy	522	80.8	N/A
	Laparoscopic appendectomy	124	19.2	
Antibiotic before surgery	No	621	96.1	0.0001
	Yes	25	3.9	
Duration of postoperative antibiotics	1 to 4 days	39	6.0	0.011
	5 days	258	39.9	
	5 to 7 days	171	26.5	
	More than 7 days	13	2.0	
Postoperative complication	Abdominal pain	1	0.2	N/A
	Hernia	1	0.2	
	Surgical-site infection	52	8.0	
	No complications	592	91.6	
Length of hospital stay	1 day or less	235	36.4	0.156
	2-3 days	346	53.6	
	More than 3 days	65	10.1	
Vomiting before	No	356	55.1	0.108
	Yes	290	44.9	
Vomiting after	No	643	99.5	N/A
	Yes	3	0.5	
Fever before	No	532	82.4	0.834
	Yes	114	17.6	
Fever after	No	637	98.6	N/A
	Yes	9	1.4	
Abdominal pain after	No	622	96.3	N/A
	Yes	24	3.7	

## Discussion

The current study analyzed 646 participants aged between less than 12 and more than 60 years, diagnosed with acute appendicitis (608, 94.1%); they were predominantly male participants, 397 (61.5%). The mean hospital stay was 2.2 days, with 582 (90.1%) of patients discharged within three days. Postoperative outcomes showed a low complication rate, with 54 (8.4%) experiencing surgical-site infections, minimal abdominal pain, and hernias. The management strategies of acute appendicitis, especially the balance between surgical intervention and antibiotic therapy, are a serious therapeutic issue. Therefore, the present study is significant in identifying the current practices related to appendicitis treatment. When comparing the study with previous literature, several major observations can be made, highlighting both similarities and differences with earlier work.

This study encompasses a wide range of participants' ages, with a mean age of 23.9 years. The sample consists of 397 male participants (61.5%) and 249 female participants (38.5%). Thus, we found a similar distribution regarding the patients’ age, consistent with the results obtained by other investigators, who also reported a higher proportion of young patients and a slightly higher number of male patients than female patients. These consistencies affirm that the patient demographic data for appendicitis have a typical baseline across diverse population categories [1,23].

In the present study, it was identified that open appendectomy was the most frequent surgical procedure, performed in 522 (80.8%) of patients, with 25 (3.9%) receiving preoperative antibiotics and a significant majority, 481 (74.5%), receiving postoperative antibiotics. This practice is contrary to what has been reported in previous studies, which have mostly considered a combination of antibiotics without any surgical involvement [21,24]. Furthermore, the identified low rates of preoperative antibiotic use did not indicate whether these patients had also received postoperative antibiotics. This is likely a target area for improvement, as their administration could be associated with a reduced incidence of surgical-site infections. Additionally, the variability in antibiotics, dosages, and treatment courses observed in the present study suggests a potential direction for revising antibiotic prophylaxis protocols in the specified clinical context. These findings can be useful in enhancing the control of future investigations and establishing more precise guidelines for the use of preoperative antibiotics and informed clinical decision-making. Likewise, this study presents a comprehensive approach to postoperative antibiotic usage, which may depend on various factors specific to each patient and their interaction with healthcare professionals. Due to the inclusion of details such as specific antibiotic regimens, routes of administration, dosages, and durations of therapy, the research data form a solid basis for understanding postoperative antibiotic practices among the members of the given population. It can be helpful for future studies, medical practice, and guidelines to understand how and when to use postoperative antibiotics to achieve optimal results and enhance patient safety.

The overall rate of postoperative complications in the present investigation was considerably low, and it was estimated that 52 (8.0%) patients developed surgical-site infections. This rate is in line with a study, which concluded that patients treated with antibiotics alone had fewer complications than those who underwent surgery [25]. Although, however, the rate of surgical-site infection was found to be higher for single antibiotic regimens than in those who did not receive antibiotics, this may be related to the empirical use of antibiotics without culture or sensitivity results, leading to bacterial resistance and consequently a higher percentage of surgical-site infections. Moreover, the length of hospital stays in this study is estimated at a mean of 2.2 days, with 582 (90.1%) patients discharged within three days. This period of hospital stay is relatively short compared to the longer hospital admissions reported previously during nonoperative management [26]. This implies effective patient handling in the postoperative period and a short recuperation period for most patients. Another aspect of the current data includes low pre- and postoperative symptoms like vomiting, fever, and abdominal pain, among others, that support the positive postoperative results. The statistical analysis revealed correlations between antibiotic regimens and the length of hospital stay, with patients not receiving antibiotics experiencing shorter stays compared to those treated with a single antibiotic. This evidence is crucial in developing strategies to promote the timely use of antibiotics and reduce recovery duration.

There is a relatively high propensity for surgical operations among patients in the current study, compared to the increasing trend in nonoperative management, and the acknowledgment of antibiotic therapy [27,28]. This variation may be associated with the accessibility or availability of certain services, as well as cultural differences that patients have regarding their treatment. Hence, there is a need to encourage patients to engage with their clinicians in a health literacy model to better understand the various treatment options.

While the current study did not directly evaluate the cost-related consequences, as supported previously [26], nonoperative management presented a cost-saving effect over time, presumably due to a decrease in inpatient length of stay. The present study provides evidence that one aspect where cost reduction could be possible is the use of preoperative antibiotics, which has declined over the years. The rates of preoperative antibiotic administration were low for this study group, suggesting the need to focus more on adherence to antibiotic guidelines set for appendicitis treatment. It also points out that surgical intervention is the favored approach, but there is an emerging trend toward higher utilization of nonoperative methods. Future studies should be conducted to identify how these regimens can be optimized, to what extent recurrence can be minimized, and which patients are most suitable for nonsurgical management.

This study has several limitations; the small sample size could have been improved by including more hospitals in Taif City, especially those from the private sector. This would have provided more insight into the use of antibiotics in patients with appendicitis, thereby increasing the accuracy of the results and offering a broader scope of clinical practice across various healthcare centers. Missing data in the medical records, precisely those related to the occurrence of adverse effects of antibiotics used (if any), in addition to missing laboratory results in several cases, such as the white blood cell count and inflammatory markers, hindered the inclusion of valuable data related to the use of antibiotics in the treatment of appendicitis in the study.

## Conclusions

Based on this study, it is reemphasized that surgical management remains dominant, and there is a low frequency of preoperative antibiotic administration. Complication rates and hospital stay lengths were low in this patient population. This study suggests that recommendations for preoperative antibiotic administration should be enhanced, and nonoperative management options should be discussed in certain patient populations, as indicated by the existing literature. In general, it is essential to focus on the rational use of antibiotics and patient-oriented methods of treatment to maximize appendicitis treatment. Future studies should focus on optimizing the course of antibiotic therapy, preventing relapses, and identifying a suitable population for nonsurgical management.
